# Impact of Sarcopenia and Pretreatment Skeletal Muscle Loss on Survival in Endometrial Cancer Patients Treated With Immune Checkpoint Inhibitors

**DOI:** 10.1111/jog.70362

**Published:** 2026-06-09

**Authors:** Komei Katayama, Nobuhisa Yoshikawa, Kaito Nonoyama, Satomi Hattori, Kosuke Yoshida, Masato Yoshihara, Satoshi Tamauchi, Akira Yokoi, Kaoru Niimi, Hiroaki Kajiyama

**Affiliations:** ^1^ Department of Obstetrics and Gynecology Nagoya University Graduate School of Medicine Nagoya Japan; ^2^ Department of Radiology Nagoya University Graduate School of Medicine Nagoya Japan

**Keywords:** endometrial neoplasms, immune checkpoint inhibitors, muscle wasting, prognosis, sarcopenia

## Abstract

**Aim:**

Sarcopenia and cancer‐related muscle wasting may affect the efficacy of immune checkpoint inhibitors (ICIs). However, their prognostic significance in endometrial cancer (EC), particularly concerning skeletal muscle status at ICI initiation and pretreatment muscle change, remains unclear. We evaluated the impact of sarcopenia at ICI initiation and pre‐ICI skeletal muscle dynamics on clinical outcomes in EC.

**Methods:**

We retrospectively analyzed 20 EC patients treated with ICIs between May 2019 and December 2024. Skeletal muscle index (SMI) was quantified using abdominal CT at the L3 level within 2 months before ICI initiation. Sarcopenia was defined as SMI < 38.5 cm^2^/m^2^. Pretreatment SMI change (ΔSMI) was calculated using baseline CT before any cancer‐directed therapy and CT immediately prior to ICI. Survival outcomes were assessed using Kaplan–Meier and Cox regression analyzes.

**Results:**

Ten patients (50%) were sarcopenic at ICI initiation and showed a trend toward poorer progression‐free survival (PFS) and overall survival (OS). Pre‐treatment muscle loss occurred in two‐thirds of patients. Patients with decreased SMI had significantly worse OS (HR 3.64, 95% CI 1.11–11.94). In subgroup analysis, SMI decline was frequent among sarcopenic patients but did not stratify survival. In contrast, among nonsarcopenic patients, greater SMI loss was associated with poorer survival, and each 1‐unit decrease in SMI significantly worsened OS (HR 1.64, 95% CI 1.05–2.63).

**Conclusions:**

Sarcopenia at ICI initiation and pretreatment skeletal muscle loss are potential predictors of poor prognosis in EC. Evaluating baseline muscle mass and its trajectory may improve risk stratification and help identify patients at higher risk of ICI resistance.

## 1 Introduction

Sarcopenia is characterized by a progressive and generalized loss of skeletal muscle mass and function, commonly associated with aging, but also frequently observed in individuals with chronic diseases, including cancer. The Asian Working Group for Sarcopenia defines the condition based on reduced muscle mass, low muscle strength, and/or impaired physical performance [1]. The prevalence of sarcopenia increases with age, affecting approximately 5%–13% of adults aged 60–70 years and up to 50% of those aged over 80 years [2]. In oncology, sarcopenia is particularly prevalent due to systemic inflammation, metabolic alterations, and treatment‐induced toxicity and has been consistently identified as an adverse prognostic factor across multiple malignancies [3, 4].

In gynecologic cancers, numerous studies have identified an association between sarcopenia and diminished chemotherapy tolerance, an increase in treatment‐related adverse events, and decreased survival rates [5, 6]. A recent meta‐analysis including more than 2400 patients with ovarian, endometrial, and cervical cancers demonstrated that sarcopenia significantly worsened both progression‐free survival (PFS) and overall survival (OS) rates [6]. Beyond being a marker of frailty, skeletal muscle is increasingly recognized as an immunologically active organ that regulates systemic inflammation, immune aging, and anti‐tumor immunity [7]. Age‐related or cancer‐induced muscle wasting contributes to chronic inflammation and impaired T‐cell function, potentially influencing the efficacy of immunotherapies [8].

ICIs have emerged as an important therapeutic option for recurrent or advanced EC, especially in tumors with microsatellite instability‐high (MSI‐H), mismatch repair deficiency (MMRd), or high tumor mutational burden. However, the clinical responses remain heterogeneous, and reliable predictive biomarkers are still lacking. Recently, attention has focused on host‐related factors, such as body composition and metabolic reserve, as potential determinants of ICI responsiveness. In particular, a recent systematic review reported that sarcopenia is likely to be a significant adverse prognostic factor in patients with solid tumors receiving ICIs, being consistently associated with both poorer treatment response and inferior survival outcomes [9]. Similarly, a high body mass index (BMI) has been associated with favorable ICI outcomes in some malignancies, whereas “sarcopenic obesity” appears to negate this advantage, highlighting the importance of qualitative rather than quantitative body composition [10, 11]. Moreover, several studies have indicated that sarcopenia may predict poor ICI response through mechanisms including impaired immune competence, heightened systemic inflammation, and cancer cachexia–associated catabolism. In particular, a comprehensive meta‐analysis by Takenaka et al. demonstrated that sarcopenia is significantly associated with reduced objective response rates and shorter survival in solid tumors treated with ICIs, underscoring its role as a clinically important host‐related prognostic factor [12].

Despite the growing evidence of other malignancies, data on sarcopenia and skeletal muscle dynamics in EC patients undergoing ICI therapy remain scarce. Importantly, most prior studies have focused on baseline sarcopenia, whereas longitudinal changes in muscle mass, reflecting cancer cachexia progression or physiological vulnerability, may better capture the host's immune and metabolic status. Whether muscle loss occurring prior to ICI initiation influences subsequent treatment outcomes has not been fully elucidated.

Therefore, the present study aimed to investigate the prognostic significance of (1) sarcopenia at the initiation of ICI therapy and (2) pretreatment changes in skeletal muscle mass in patients with EC. Clarifying the impact of host body composition may contribute to improved risk stratification and personalized immunotherapy strategies for this population.

## 2 Patients and Methods

### 2.1 Study Design and Patients

This retrospective observational study included patients with histologically confirmed EC who received ICI therapy at Nagoya University Hospital between May 2019 and December 2024. All patients who underwent abdominal computed tomography (CT) within 2 months before ICI initiation and had complete clinical follow‐up data were included. No patients were excluded because of insufficient imaging or missing clinical data.

### 2.2 Treatment

Patients received either pembrolizumab monotherapy or combination therapy with pembrolizumab and lenvatinib according to clinical indications and physician discretion. Treatment cycles, dosing schedules, and prior systemic therapies were obtained from the patients' medical records.

### 2.3 Assessment of Skeletal Muscle Mass

Skeletal muscle mass was quantified using axial CT images at the level of the third lumbar vertebra (L3). CT‐based assessment of skeletal muscle area at the L3 vertebral level is widely accepted as a validated surrogate marker of whole‐body skeletal muscle mass and has been commonly used in oncologic sarcopenia research [3, 13]. Skeletal muscle area (SMA) was measured using SYNAPSE VINCENT (Fujifilm Co., Tokyo, Japan), a semiautomated body composition analysis software. SMA included the psoas, erector spinae, quadratus lumborum, rectus abdominis, and abdominal wall muscles. Skeletal muscle index (SMI) was calculated as SMA (cm^2^) divided by height squared (m^2^). Sarcopenia was defined as SMI < 38.5 cm^2^/m^2^, as described in previous reports [3].

### 2.4 Evaluation of Pretreatment Muscle Change

To assess longitudinal changes, SMI was evaluated at two time points:

1. baseline CT obtained before any cancer‐directed therapy, and
2. CT performed immediately before ICI initiation.
  ΔSMI was calculated as SMI (before‐ICI) – SMI (baseline). Patients were categorized into the decreased‐SMI group (ΔSMI < 0) and nondecreased group.

### 2.5 Clinical Variables and Outcomes

Demographic and clinical characteristics, including age, BMI, histological subtype, FIGO stage, and treatment history, were collected from medical records. Mismatch repair (MMR)/microsatellite instability (MSI) status was also collected when available. MMR status was assessed by immunohistochemistry for MLH1, MSH2, MSH6, and PMS2, and/or MSI status was determined by PCR‐based testing. Tumors were classified as MMR‐deficient (dMMR)/MSI‐high (MSI‐H) or MMR‐proficient (pMMR)/microsatellite stable (MSS). The primary outcomes were PFS and OS. PFS was defined as the interval from ICI initiation to radiologic or clinical disease progression or death. OS was defined as the interval from ICI initiation to death from any cause.

### 2.6 Statistical Analysis

Continuous variables were compared using the Student's *t*‐test or the Mann–Whitney U test, and categorical variables were compared using the *χ* ^2^ test or Fisher's exact test. Survival curves were estimated using the Kaplan–Meier method and compared using the log‐rank test. Hazard ratios (HRs) and 95% confidence intervals (CIs) were calculated using Cox proportional hazards model. Statistical significance was defined as *p* < 0.05. All analyzes were performed using GraphPad Prism v10.6.1.

## 3 Results

### 3.1 Patient Characteristics

The overall baseline demographic and clinical characteristics stratified by sarcopenia status are summarized in Table 1. Twenty patients with EC treated with ICIs were included in the study. The mean age of the cohort was 62.30 ± 7.46 years, and the mean BMI was 21.94 ± 3.59 kg/m^2^. The overall mean SMI was 37.87 ± 7.04 cm^2^/m^2^. At the initiation of ICI therapy, 10 patients (50%) met the criteria for sarcopenia (SMI < 38.5 cm^2^/m^2^). The mean SMI was significantly lower in the sarcopenia group than in the nonsarcopenia group (32.33 ± 3.92 vs. 43.42 ± 4.56 cm^2^/m^2^, *p* < 0.001). Other baseline characteristics, including tumor histology, FIGO stage, presence or absence of lymphovascular space invasion (LVSI), and prior treatment history, did not differ significantly between the two groups. Similarly, there were no significant differences in the ICI regimen (pembrolizumab monotherapy vs. pembrolizumab and lenvatinib), treatment duration, or number of prior therapy lines before ICI initiation.

**TABLE 1:** Patient characteristics at the time of ICI initiation, stratified by sarcopenia status.

Variables	Total (*n* = 20)	Sarcopenia (*n* = 10)	Non—sarcopenia (*n* = 10)	*p*
Age (years), mean ± SD	62.30 ± 7.46	61.10 ± 6.98	63.50 ± 8.10	0.49
BMI (kg/m^2^), mean ± SD	21.94 ± 3.59	20.56 ± 3.43	23.31 ± 3.36	0.09
SMI (cm^2^/m^2^), mean ± SD	37.87 ± 7.04	32.33 ± 3.92	43.42 ± 4.56	< 0.001
Histological type, *n* (%)				0.85
Endometrioid G1/2	6 (30%)	2 (20%)	4 (40%)	
Endometrioid G3	7 (35%)	4 (40%)	3 (30%)	
Carcinosarcoma	5 (25%)	3 (30%)	2 (20%)	
Others	2 (10%)	1 (10%)	1 (10%)	
Stage (FIGO 2018), *n* (%)				1
I	8 (40%)	4 (40%)	4 (40%)	
II	0 (0%)	0 (0%)	0 (0%)	
III	7 (35%)	3 (30%)	4 (40%)	
IV	5 (25%)	3 (30%)	2 (20%)	
LVSI, *n* (%)				1
(−)	6 (30%)	3 (30%)	3 (30%)	
(+)	14 (70%)	7 (70%)	7 (70%)	
MMR/MSI status, *n* (%)				1
dMMR/MSI‐H	7 (35%)	4 (40%)	3 (30%)	
pMMR/MSS	9 (45%)	4 (40%)	5 (50%)	
Not assessed	4 (20%)	2 (20%)	2 (20%)	
Systematic lymphadenectomy, *n* (%)				1
No	9 (45%)	4 (40%)	5 (50%)	
Yes	11 (55%)	6 (60%)	5 (50%)	
Prior chemotherapy regimens, *n* (%)				0.3
1	15 (75%)	9 (90%)	6 (60%)	
≥ 2	5 (25%)	1 (10%)	4 (40%)	
TFI, *n* (%)				0.14
< 6 m	14 (70%)	9 (90%)	5 (50%)	
≥ 6 m	6 (30%)	1 (10%)	5 (50%)	
ICI regimen, *n* (%)				0.63
Pembrolizumab alone	6 (30%)	4 (40%)	2 (20%)	
Pembrolizumab + Lenvatinib	14 (70%)	6 (60%)	8 (80%)	
ICI treatment duration (months), median (range)	11.67 (0.93–43.17)	10.97 (1.57–43.17)	12.37 (0.93–24.13)	0.76
Pembrolizumab cycles, median (range)	10 (1–37)	10 (2–37)	15 (1–27)	0.72

Abbreviations: 6 m, 6 months; BMI, body mass index; dMMR, MMR‐deficient; FIGO, International Federation of Gynecology and Obstetrics staging system; ICI, immune checkpoint inhibitor; LVSI, lymphovascular space invasion; MMR, mismatch repair; MSI, microsatellite instability; MSI‐H, MSI‐High; MSS, microsatellite stable; Others, serous carcinoma/clear cell carcinoma/mixed carcinoma/dedifferentiated‐carcinoma/undifferentiated carcinoma; pMMR, MMR‐proficient; SMI, skeletal muscle index; TFI, treatment‐free interval.

### 3.2 Survival Outcomes According to Sarcopenia Status

**FIGURE 1:**
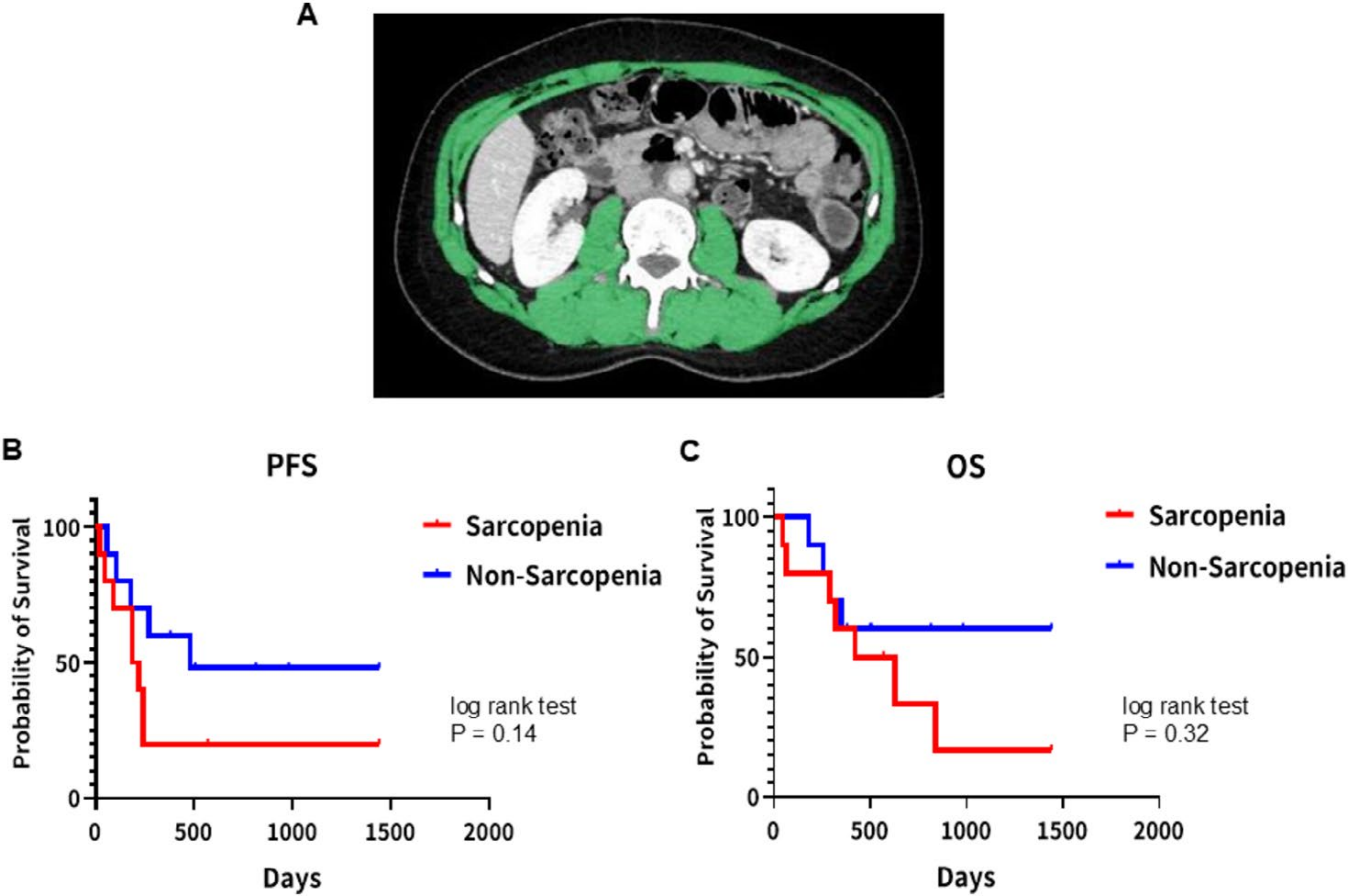
Assessment of skeletal muscle index (SMI) and survival outcomes according to sarcopenia status. (A) Representative axial CT image at the L3 level showing segmented skeletal muscle area (green). The skeletal muscle area (SMA) is highlighted in green and includes the psoas, erector spinae, quadratus lumborum, transversus abdominis, internal oblique, external oblique, and rectus abdominis muscles. SMA (cm^2^) was quantified using semi‐automated segmentation software (SYNAPSE VINCENT), and SMI (cm^2^/m^2^) was calculated by normalizing SMA to the patient's height squared. This L3 cross‐sectional area is widely recognized as a validated surrogate marker for whole‐body skeletal muscle mass. (B) Progression‐free survival of patients with versus without sarcopenia. (C) Overall survival of patients with versus without sarcopenia. OS, overall survival; PFS, progression‐free survival.

Skeletal muscle mass was assessed using axial CT images at the level of the third lumbar vertebra (L3), where the skeletal muscle area was segmented and quantified to calculate the SMI. An example of an L3 cross‐sectional image used for muscle measurement is shown in Figure 1A. Patients with sarcopenia exhibited poorer survival outcomes than those without sarcopenia. PFS was shorter in the sarcopenia group, although the difference was not statistically significant (HR 2.35, 95% CI 0.76–7.31; log‐rank *p* = 0.14). A similar trend was observed for OS (HR 1.84, 95% CI 0.56–6.00; log‐rank *p* = 0.32). Kaplan–Meier curves for PFS and OS according to sarcopenia status are presented in Figure 1B,C.

### 3.3 Pretreatment Skeletal Muscle Mass Change

**FIGURE 2:**
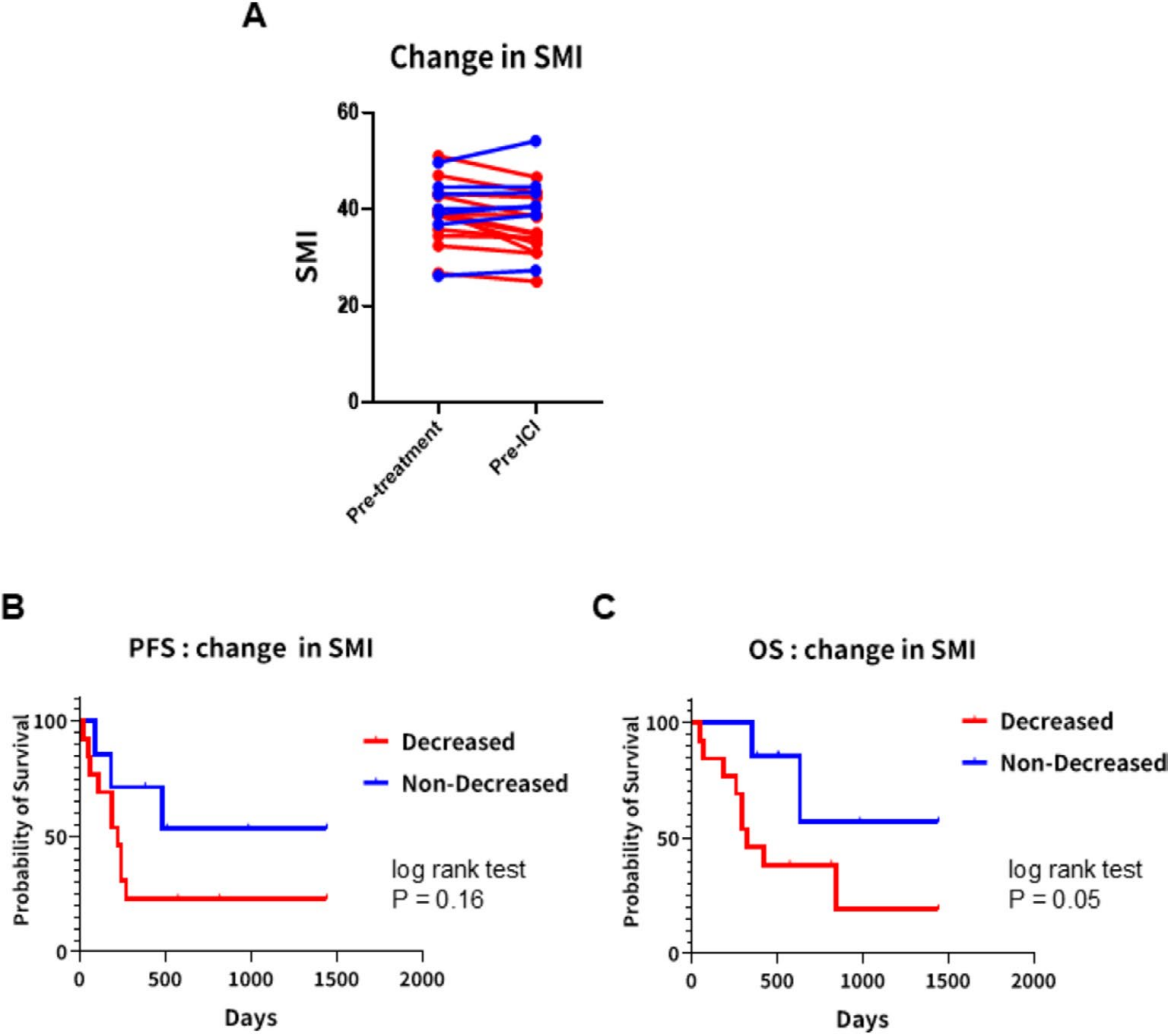
Pretreatment skeletal muscle mass change and survival outcomes according to SMI trajectory. (A) Individual changes in SMI from baseline CT (before any cancer‐directed therapy) to the CT immediately prior to ICI initiation. Red lines indicate patients with decreased SMI (ΔSMI < 0), and blue lines indicate patients without SMI decrease (ΔSMI ≥ 0). (B) PFS stratified by SMI decrease versus nondecrease. (C) OS stratified by SMI decrease versus nondecrease. ICI, immune checkpoint inhibitor; OS, overall survival; PFS, progression‐free survival; SMI, skeletal muscle index.

Longitudinal changes in skeletal muscle mass were assessed by comparing the SMI at two time points: (1) baseline CT obtained before any cancer‐directed therapy and (2) CT acquired immediately before ICI initiation. Based on the ΔSMI values, patients were categorized into the decreased‐SMI group (*n* = 13) and the non‐decreased group (*n* = 7). Figure 2A illustrates the individual SMI trajectories from baseline to immediately before ICI initiation. Two‐thirds of the patients experienced a decrease in SMI, with a median ΔSMI of −1.20 cm^2^/m^2^ (IQR −3.85 to 0.36). The median ΔSMI was −3.52 (IQR −4.37 to −1.58) cm^2^/m^2^ in the decreased SMI group and 1.09 (IQR 0.36 to 1.78) cm^2^/m^2^ in the nondecreased SMI group (*p* < 0.001). The baseline demographic and clinical characteristics, including age, BMI, tumor histology, FIGO stage, presence of LVSI, prior treatment history, ICI regimen, and treatment duration, did not differ significantly between the decreased SMI and nondecreased SMI groups (Table 2).

**TABLE 2:** Comparison of baseline characteristics between the decreased‐SMI and nondecreased‐SMI groups.

Variables	Decreased‐SMI (*n* = 13)	Nondecreased‐SMI (*n* = 7)	*p*
Age (years), mean ± SD	60.92 ± 7.54	64.86 ± 7.13	0.27
Histological type, *n* (%)			0.94
Endometrioid G1/2	4 (31%)	2 (29%)	
Endometrioid G3	5 (38%)	2 (29%)	
Carcinosarcoma	3 (23%)	2 (29%)	
Others	1 (8%)	1 (13%)	
Stage (FIGO 2018), *n* (%)			0.64
I	6 (46%)	2 (29%)	
II	0 (0%)	0 (0%)	
III	3 (23%)	4 (57%)	
IV	4 (31%)	1 (14%)	
LVSI, *n* (%)			1
(−)	4 (31%)	2 (29%)	
(+)	9 (69%)	5 (71%)	
MMR/MSI status, *n* (%)			0.72
dMMR/MSI‐H	5 (38%)	2 (29%)	
pMMR/MSS	5 (38%)	4 (57%)	
Not assessed	3 (24%)	1 (14%)	
Prior chemotherapy regimens, *n* (%)			0.29
1	11 (85%)	4 (57%)	
≥ 2	2 (15%)	3 (43%)	
TFI, *n* (%)			0.61
< 6 m	10 (77%)	4 (57%)	
≥ 6 m	3 (23%)	3 (43%)	
ICI regimen, *n* (%)			0.35
Pembrolizumab alone	5 (38%)	1 (14%)	
Pembrolizumab + Lenvatinib	8 (62%)	6 (86%)	
ICI treatment duration (months), median (range)	10.94 (0.93–43.17)	13.02 (3.20–24.13)	0.67
ΔSMI (cm^2^/m^2^), median (IQR)	−3.52 (−4.37 to −1.58)	1.09 (0.36–1.78)	< 0.001
Interval between CT scans (months), median (IQR)	10 (8–16)	45 (20.5–126)	0.07

Abbreviations: ΔSMI, change in skeletal muscle index between baseline and pre‐ICI; dMMR, MMR‐deficient; FIGO, International Federation of Gynecology and Obstetrics; ICI, immune checkpoint inhibitor; IQR, interquartile range; LVSI, lymphovascular space invasion; MMR, mismatch repair; MSI, microsatellite instability; MSI‐H, MSI‐High; MSS, microsatellite stable; Others, serous carcinoma/clear cell carcinoma/mixed carcinoma/dedifferentiated carcinoma/undifferentiated carcinoma; pMMR, MMR‐proficient; Range, minimum–maximum values; SMI, skeletal muscle index; TFI, treatment‐free interval.

The median interval between the two CT examinations used for the ΔSMI calculation was 13 months (IQR 8.75–89) for the entire cohort, 10 months (IQR 8–16) for the decreased‐SMI group, and 45 months (IQR 20.5–126) for the non‐decreased group, with no statistically significant difference (*p* = 0.074). Patients with decreased SMI demonstrated significantly shorter OS (HR 3.64, 95% CI 1.11–11.94; log‐rank *p* = 0.05) and a trend toward shorter PFS (HR 2.42, 95% CI 0.80–7.26; log‐rank *p* = 0.16) than those without SMI decline (Figure 2B,C).

### 3.4 Subgroup Analysis by Sarcopenia Status

To further explore the prognostic relevance of skeletal muscle dynamics, subgroup analyzes were conducted separately for the sarcopenia (*n* = 10) and nonsarcopenia (*n* = 10) groups. Among patients with sarcopenia, 9 of 10 patients (90%) exhibited a decrease in SMI, whereas only one patient showed no decrease. In contrast, in the non‐sarcopenia group, 4 of 10 patients (40%) showed decreased SMI and six patients (60%) showed no decline. The median ΔSMI (IQR) was −2.79 (−4.34 to −1.62) in the sarcopenia group and 0.24 (−0.63 to 1.30) in the non‐sarcopenia group (Table 3).

**TABLE 3:** Comparison of ΔSMI between sarcopenia and nonsarcopenia groups.

Variables	Sarcopenia (*n* = 10)	Nonsarcopenia (*n* = 10)
SMI decrease, *n* (%)	9 (90%)	4 (40%)
SMI nondecrease, *n* (%)	1 (10%)	6 (60%)
ΔSMI, median (IQR)	−2.79 (−4.34 to −1.62)	0.24 (−0.63 to 1.30)

Abbreviations: ΔSMI, change in skeletal muscle index from baseline to pre‐ICI assessment; IQR = interquartile range; SMI = skeletal muscle index.

**FIGURE 3:**
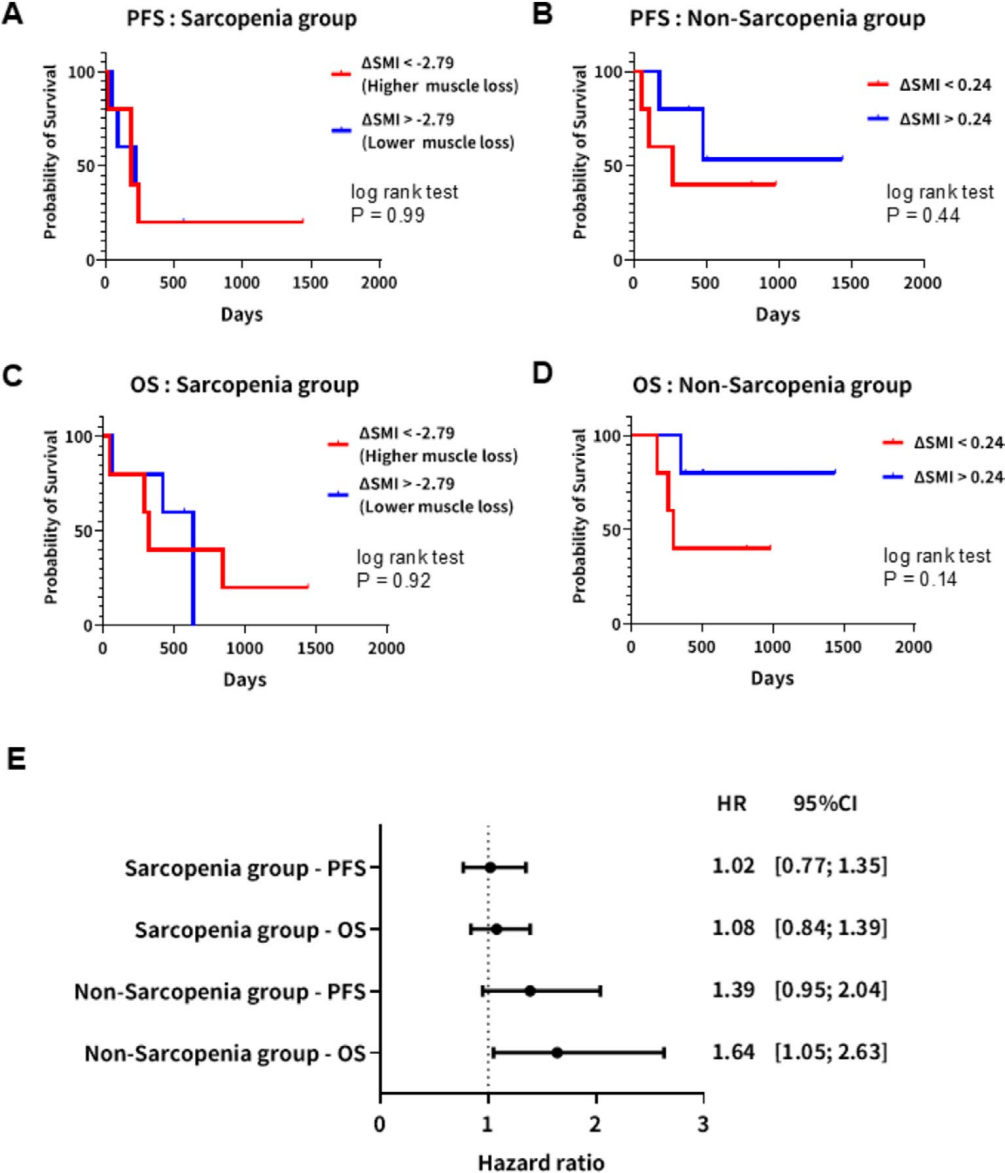
Subgroup analyzes of survival outcomes according to pretreatment skeletal muscle mass change (ΔSMI). (A) Kaplan–Meier curves for PFS in the sarcopenia group stratified by median ΔSMI (−2.79). (B) PFS curves in the nonsarcopenia group stratified by median ΔSMI (0.24). (C) OS curves in the sarcopenia group stratified by median ΔSMI. (D) OS curves in the nonsarcopenia group stratified by median ΔSMI. (E) Forest plot summarizing hazard ratios for each subgroup associated with a 1‐unit decrease in SMI. OS, overall survival; PFS, progression‐free survival; SMI, skeletal muscle index.

Kaplan–Meier survival analyzes demonstrated no significant association between ΔSMI and survival outcomes within the sarcopenia group: PFS (log‐rank *p* = 0.99) and OS (log‐rank *p* = 0.92). In the nonsarcopenia group, patients with greater SMI loss tended to have poorer survival, although the results did not reach statistical significance: PFS (log‐rank *p* = 0.44) and OS (log‐rank *p* = 0.14). Cox proportional hazards models were applied to quantify the magnitude of the risk associated with continuous changes in SMI. In the sarcopenia group, each 1‐unit decrease in SMI was not significantly associated with PFS (HR 1.02, 95% CI 0.77–1.35) or OS (HR 1.08, 95% CI 0.84–1.39). In the non‐sarcopenia group, however, a decreasing SMI demonstrated a stronger effect on survival, particularly OS: PFS (HR 1.39, 95% CI 0.95–2.04) and OS (HR 1.64, 95% CI 1.05–2.63). These findings are shown in Figure 3.

## 4 Discussion

In this study, we demonstrated that skeletal muscle status at the time of ICI initiation as well as pre‐treatment longitudinal skeletal muscle loss were associated with clinical outcomes in patients with EC undergoing immunotherapy. Patients with sarcopenia at ICI initiation tended to show worse survival, while pre‐ICI SMI decline was identified as an additional predictor of poor outcomes, particularly among those who were not sarcopenic at the treatment initiation. These findings highlight the importance of evaluating not only static muscle mass at the start of treatment but also muscle trajectory during the pre‐ICI period, supporting the hypothesis that skeletal muscle mass and its trajectory reflect host physiological and immunological reserves at the time of therapy, which can influence therapeutic efficacy.

The skeletal muscle is increasingly recognized not only as a locomotive organ but also as an immunologically active tissue. Muscle tissue secretes myokines that modulate systemic inflammation, immune homeostasis, and T‐cell metabolism, potentially influencing anti‐tumor immunity [7]. Low muscle mass or cachexia can be associated with chronic inflammation, metabolic dysregulation, and impaired immune competence—factors that may blunt the efficacy of ICIs. Indeed, a recent review concluded that low muscle mass/cachexia detrimentally affects immunotherapy outcomes in many solid tumors [14]. Therefore, muscle wasting prior to ICI therapy may indicate a state of reduced immunologic reserve.

Importantly, even among patients with sarcopenia, some cases respond well to ICI; thus, low muscle mass alone should not be used to exclude patients from ICI therapy. Our data support this: in the sarcopenia subgroup, ΔSMI (further loss) did not significantly stratify survival (PFS, OS), suggesting that some patients with sarcopenia may still benefit from treatment. This argues against the use of sarcopenia or muscle loss as strict exclusion criteria for ICI. Instead, we propose that muscle mass assessment (and its changes) be used to inform supportive care and intervention rather than as a gatekeeper for ICI. In other words, rather than “if low muscle mass → avoid ICI,” a more constructive approach is “if low or declining muscle mass → proactively intervene to preserve muscle and maximize ICI benefit.”

Emerging evidence suggests that interventions combining nutritional support and physical activity may help maintain or even improve muscle mass in cancer patients, even during systemic therapy. For example, resistance exercise has been recognized as a promising strategy for improving muscle strength, body composition, and physical function in cancer survivors, potentially mitigating sarcopenia [15]. Similarly, specialized nutritional support during chemotherapy has been shown to help preserve muscle function and lean body mass [16]. Although data on ICI‐treated patients are limited, the general principle of preserving lean mass may apply and improve immunotherapy outcomes by maintaining the patient's metabolic/immune reserve. Accordingly, for patients scheduled to receive ICI, particularly those with sarcopenia or at risk for muscle loss (e.g., after prior chemotherapy), incorporating a prehabilitation or cachexia‐directed strategy (nutrition counseling, resistance training, and physical therapy) could be advantageous. This aligns with the broader oncologic cachexia management strategies [17].

Cytotoxic chemotherapy used in EC may accelerate muscle wasting not only through its direct catabolic effects but also indirectly via treatment‐related adverse events, such as nausea, appetite loss, fatigue, and reduced physical activity. These toxicities can cumulatively lead to decreased dietary intake and lower energy expenditure, thereby worsening sarcopenia or precipitating further muscle loss in vulnerable patients. In addition, patients who received multiple lines of prior chemotherapy may have experienced greater cumulative toxicity and physiological burden. Therefore, the observed association between pre‐treatment SMI decline and survival outcomes may be partially confounded by prior treatment exposure, and a causal relationship cannot be definitively established in this study. In individuals with borderline muscle mass or those who experience substantial chemotherapy‐induced toxicity, an earlier transition to immunotherapy—when clinically appropriate—may help prevent additional deterioration in muscle function and preserve physiological reserve. Although our data do not directly test this strategy, the observed prognostic impact of pretreatment muscle loss supports the need for careful consideration of ICI timing in high‐risk patients.

This study has several limitations. First, the sample size was small (*n* = 20), which reduced the statistical power and may have limited generalizability. Furthermore, the small sample size increases the risk of Type I errors. Therefore, the findings should be interpreted with caution, and further validation in larger multicenter cohorts is warranted to confirm these findings. Second, this was a retrospective, single‐center study with the potential for selection bias. Third, although we assessed SMI at two time points, the CT intervals varied widely among patients, which may have introduced measurement bias. Fourth, we did not systematically collect data on nutritional intake, physical activity, or inflammatory markers; thus, we cannot definitively attribute muscle loss to cachexia, disuse, or other causes. In addition, MMR/MSI status was not available in all patients because molecular testing was not routinely performed during the earlier study period. Finally, intervention efficacy (e.g., resistance exercise or nutritional support) was not studied here; thus, recommendations remain speculative and need confirmation in prospective trials.

To build on our findings, prospective studies are needed that (1) monitor muscle mass longitudinally in ICI‐treated patients, (2) integrate interventions (nutrition, exercise, cachexia‐directed therapies), (3) collect immunologic biomarkers (inflammatory cytokines, T‐cell function), and (4) evaluate whether muscle preservation improves ICI response and survival. The development of standardized protocols for prehabilitation and cachexia management in the context of immunotherapy would be valuable.

In summary, both sarcopenia at ICI initiation and pre‐treatment skeletal muscle loss were associated with poor outcomes in patients with EC treated with ICIs. Muscle trajectory may serve as a valuable host‐related marker for identifying individuals with declining physiological and immunological reserves. Importantly, sarcopenia should not deter clinicians from offering ICI therapy, as therapeutic benefits can still be achieved in these patients. These findings highlight the need for proactive strategies to preserve skeletal muscles before and during treatment. Prospective studies incorporating nutritional support, structured exercise, and other host‐directed interventions are required to optimize immunotherapy outcomes.

## Author Contributions

**Kaito Nonoyama:** software, methodology, validation. **Komei Katayama:** writing – original draft, conceptualization, methodology, formal analysis, validation. **Nobuhisa Yoshikawa:** writing – review and editing, supervision, methodology. **Kaoru Niimi:** investigation, validation. **Kosuke Yoshida:** investigation, validation. **Satoshi Tamauchi:** investigation, validation. **Satomi Hattori:** investigation, validation. **Masato Yoshihara:** investigation, validation. **Hiroaki Kajiyama:** supervision, investigation, validation. **Akira Yokoi:** investigation, validation.

## Disclosure

The authors have nothing to report.

## Ethics Statement

This study was approved by the Institutional Review Board of Nagoya University Hospital (approval number: 2013‐0078). The study was conducted in accordance with the Declaration of Helsinki and applicable local regulations.

## Consent

The requirement for informed consent was waived due to the retrospective design, and an opt‐out policy was implemented.

## Conflicts of Interest

The authors declare no conflicts of interest.

## Data Availability

The datasets generated and/or analyzed during the current study are not publicly available because they contain potentially identifiable patient‐level clinical information, including imaging‐derived body composition parameters, treatment details, and survival outcomes of patients with endometrial cancer treated with immune checkpoint inhibitors. Public sharing of these individual‐level data was not included in the consent process or institutional ethics approval for this retrospective study. Data may be made available from the corresponding author upon reasonable request, subject to approval by the relevant institutional ethics committee and in accordance with applicable privacy regulations and institutional policies.

## References

[1] L. K. Chen , L. K. Liu , J. Woo , et al., “Sarcopenia in Asia: Consensus Report of the Asian Working Group for Sarcopenia,” Journal of the American Medical Directors Association 15 (2014): 95–101. 24461239 10.1016/j.jamda.2013.11.025

[2] A. J. Cruz‐Jentoft , J. P. Baeyens , J. M. Bauer , et al., “Sarcopenia: European Consensus on Definition and Diagnosis: Report of the European Working Group on Sarcopenia in Older People,” Age and Ageing 39 (2010): 412–423. 20392703 10.1093/ageing/afq034 PMC2886201

[3] C. M. Prado , J. R. Lieffers , M. C. LJ , et al., “Prevalence and Clinical Implications of Sarcopenic Obesity in Patients With Solid Tumours of the Respiratory and Gastrointestinal Tracts: A Population‐Based Study,” Lancet Oncology 9 (2008): 629–635. 18539529 10.1016/S1470-2045(08)70153-0

[4] S. S. Shachar , G. R. Williams , H. B. Muss , and T. F. Nishijima , “Prognostic Value of Sarcopenia in Adults With Solid Tumours: A Meta‐Analysis and Systematic Review,” European Journal of Cancer 57 (2016): 58–67. 26882087 10.1016/j.ejca.2015.12.030

[5] I. J. G. Rutten , J. Ubachs , R. F. P. M. Kruitwagen , et al., “The Influence of Sarcopenia on Survival and Surgical Complications in Ovarian Cancer Patients Undergoing Primary Debulking Surgery,” European Journal of Surgical Oncology 43 (2017): 717–724. 28159443 10.1016/j.ejso.2016.12.016

[6] E. R. Allanson , Y. Peng , A. Choi , S. Hayes , M. Janda , and A. Obermair , “A Systematic Review and Meta‐Analysis of Sarcopenia as a Prognostic Factor in Gynecological Malignancy,” International Journal of Gynecological Cancer 30 (2020): 1791–1797. 32747410 10.1136/ijgc-2020-001678

[7] C. Nelke , R. Dziewas , J. Minnerup , S. G. Meuth , and T. Ruck , “Skeletal Muscle as Potential Central Link Between Sarcopenia and Immune Senescence,” eBioMedicine 49 (2019): 381–388. 31662290 10.1016/j.ebiom.2019.10.034 PMC6945275

[8] N. Zhang , L. Zhai , R. M. Y. Wong , et al., “Harnessing Immunomodulation to Combat Sarcopenia: Current Insights and Possible Approaches,” Immunity & Ageing 21 (2024): 55. 39103919 10.1186/s12979-024-00458-9 PMC11299351

[9] S. Li , T. Wang , G. Tong , X. Li , D. You , and M. Cong , “Prognostic Impact of Sarcopenia on Clinical Outcomes in Malignancies Treated With Immune Checkpoint Inhibitors: A Systematic Review and Meta‐Analysis,” Frontiers in Oncology 11 (2021): 726257. 34513704 10.3389/fonc.2021.726257 PMC8427761

[10] J. H. Lee , D. Kang , J. S. Ahn , E. Guallar , J. Cho , and H. Y. Lee , “Obesity Paradox in Patients With Non‐Small Cell Lung Cancer Undergoing Immune Checkpoint Inhibitor Therapy,” Journal of Cachexia, Sarcopenia and Muscle 14 (2023): 2898–2907. 37964713 10.1002/jcsm.13367 PMC10751411

[11] G. S. Naik , S. S. Waikar , A. E. W. Johnson , et al., “Complex Inter‐Relationship of Body Mass Index, Gender and Serum Creatinine on Survival: Exploring the Obesity Paradox in Melanoma Patients Treated With Checkpoint Inhibition,” Journal for Immunotherapy of Cancer 7 (2019): 89. 30922394 10.1186/s40425-019-0512-5 PMC6440018

[12] Y. Takenaka , R. Oya , N. Takemoto , and H. Inohara , “Predictive Impact of Sarcopenia in Solid Cancers Treated With Immune Checkpoint Inhibitors: A Meta‐Analysis,” Journal of Cachexia, Sarcopenia and Muscle 12 (2021): 1122–1135. 34337889 10.1002/jcsm.12755 PMC8517360

[13] M. Mourtzakis , C. M. M. Prado , J. R. Lieffers , T. Reiman , L. J. McCargar , and V. E. Baracos , “A Practical and Precise Approach to Quantification of Body Composition in Cancer Patients Using Computed Tomography Images Acquired During Routine Care,” Applied Physiology, Nutrition, and Metabolism 33 (2008): 997–1006. 10.1139/H08-075 18923576

[14] Y. Deng , L. Zhao , X. Huang , Y. Zeng , Z. Xiong , and M. Zuo , “Contribution of Skeletal Muscle to Cancer Immunotherapy: A Focus on Muscle Function, Inflammation, and Microbiota,” Nutrition 105 (2023): 111829. 36265324 10.1016/j.nut.2022.111829

[15] M. K. Jang , C. Park , L. Tussing‐Humphreys , B. Fernhall , S. Phillips , and A. Z. Doorenbos , “The Effectiveness of Sarcopenia Interventions for Cancer Patients Receiving Chemotherapy: A Systematic Review and Meta‐Analysis,” Cancer Nursing 46 (2023): E81–E90. 34054070 10.1097/NCC.0000000000000957 PMC8627517

[16] L. A. Wijler , D. A. E. Raats , S. G. Elias , et al., “Specialized Nutrition Improves Muscle Function and Physical Activity Without Affecting Chemotherapy Efficacy in C26 Tumour‐Bearing Mice,” Journal of Cachexia, Sarcopenia and Muscle 12 (2021): 796–810. 33956410 10.1002/jcsm.12703 PMC8200448

[17] E. Papadopoulos , B. A. Irving , J. C. Brown , et al., “Sarcopenia and Cachexia in Older Patients With Cancer: Pathophysiology, Diagnosis, Impact on Outcomes, and Management Strategies,” Drugs and Aging 42 (2025): 1113–1142. 41060616 10.1007/s40266-025-01252-y PMC12660466

